# DeepBiomarker2: Prediction of alcohol and substance use disorder risk in post-traumatic stress disorder patients using electronic medical records and multiple social determinants of health

**DOI:** 10.21203/rs.3.rs-2949487/v1

**Published:** 2023-05-25

**Authors:** Oshin Miranda, Peihao Fan, Xiguang Qi, Haohan Wang, M Daniel Brannock, Thomas Kosten, Neal David Ryan, Levent Kirisci, LiRong Wang

**Affiliations:** University of Pittsburgh; University of Pittsburgh; University of Pittsburgh; University of Illinois Urbana-Champaign; RTI International; Baylor College of Medicine; University of Pittsburgh; University of Pittsburgh; University of Pittsburgh

**Keywords:** Post traumatic stress disorder, alcohol and substance use disorder, social determinants of health, deep learning, biomarker identification

## Abstract

**Introduction:**

Prediction of high-risk events amongst patients with mental disorders is critical for personalized interventions. In our previous study, we developed a deep learning-based model, DeepBiomarker by utilizing electronic medical records (EMR) to predict the outcomes of patients with suicide-related events in post-traumatic stress disorder (PTSD) patients.

**Methods:**

We improved our deep learning model to develop DeepBiomarker2 through data integration of multimodal information: lab tests, medication use, diagnosis, and social determinants of health (SDoH) parameters (both individual and neighborhood level) from EMR data for outcome prediction. We further refined our contribution analysis for identifying key factors. We applied DeepBiomarker2 to analyze EMR data of 38,807 patients from University of Pittsburgh Medical Center diagnosed with PTSD to determine their risk of developing alcohol and substance use disorder (ASUD).

**Results:**

DeepBiomarker2 predicted whether a PTSD patient will have a diagnosis of ASUD within the following 3 months with a c-statistic (receiver operating characteristic AUC) of 0·93. We used contribution analysis technology to identify key lab tests, medication use and diagnosis for ASUD prediction. These identified factors imply that the regulation of the energy metabolism, blood circulation, inflammation, and microbiome is involved in shaping the pathophysiological pathways promoting ASUD risks in PTSD patients. Our study found protective medications such as oxybutynin, magnesium oxide, clindamycin, cetirizine, montelukast and venlafaxine all have a potential to reduce risk of ASUDs.

**Discussion:**

DeepBiomarker2 can predict ASUD risk with high accuracy and can further identify potential risk factors along with medications with beneficial effects. We believe that our approach will help in personalized interventions of PTSD for a variety of clinical scenarios.

## 1. Introduction

Posttraumatic stress disorder (PTSD) and alcohol and substance use disorder (ASUD) often co-occur, with an estimated prevalence of ASUD amongst individuals with PTSD of 46% in the United States alone^1^. Patients with ASUD experience higher rates of PTSD, with the highest rates reported in patients with both alcohol and drug use disorders^2^. Current literature has enlisted mechanisms that may explain this co-occurrence: (1) ASUD could exacerbate the risk of developing PTSD, as patients tend to lead a high risk lifestyle which increases the chances of being exposed to or experience traumatic event (e.g.: sexual assault under the influence of substances)^3^; (2) PTSD development can precede ASUD as patients use substances to self-medicate their PTSD symptoms^4^; (3) Genetic influences on the onset, maintenance, or etiology of both disorders^4, 5^; and (4) Multiple aspects of well-being such as psychosocial risk and protective factors extracted from social determinants of health (SDOH)^6^ could be related to a shared underlying factor affecting the overall quality of life. While various studies suggest that both PTSD and ASUD share common dysfunctions pertaining to numerous biological systems, it is paramount to determine multiple risk and beneficial factors that are responsible for development of ASUD in PTSD, and this can be achieved via application of novel analytic technologies to data-mine EMR data from these patients. In addition, current treatment of PTSD and ASUD is limited^7^.

Social determinants of health (SDoH) are “conditions or environments in which people are born, grow, live, work, and age,”^8^. Five key SDoH domains that have significant impacts on human health are: (1) economic stability, (2) education, (3) health and health care, (4) neighborhood and built environment, and (5) social and community context^9^. These complex, integrated, and overlapping social and economic systems in turn are responsible for most health inequalities and poor health outcomes existing today. Electronic medical records (EMRs) are an important component of clinical practice and documentation. However, a major limitation of EMRs is the lack of reliable SDoH information and documentation, which is strongly associated with mental health^10^. EMRs collect clinical information such as diagnosis, medication use, laboratory test results, vital signs, procedures, and other data in a systemic fashion. Other nonclinical determinants of health such as age, race, ethnicity is collected in a structured EMR format. Current studies have linked a variety of SDoH parameters such as neighborhood socioeconomic status (nSES) indicators with disease risk factors to improve the accuracy of risk prediction models^11, 12^. By extrapolating information obtained from both conventional (e.g., EMR sources) and non-conventional sources (e.g. SDoH databases, clinical EMR notes, census data), one can use this “big data” for risk prediction and development of interventions to improve multiple clinical outcomes especially focusing on high risk patients such as patients with multiple comorbidities. However, to the best of our knowledge, few studies assess the importance of this wide range of multimodal information such as diagnosis, medication use, laboratory test results, individual level SDoH indicators (e.g., race, age, gender, etc.) and neighborhood level SDoH indicators (e.g., nSES index, etc.) in the prediction of outcomes of patients with mental disorders.

Deep learning/data mining algorithms translate “big data” into valuable information for hypothesis generation through deep hierarchical feature construction to capture long-range dependencies in EMR data^13^. Deep learning techniques learn certain features directly from the data itself, without any human guidance, thus allowing for automatic discovery of latent data relationships that might otherwise be hidden^14^. By analyzing the EMR of PTSD patients, we can find risk factors or medications that can have the potential to reshape the trajectory of disease progression and we can then use these findings to design better treatment for those patients. In this study, we improved our previous deep learning based Deepbiomarker model to develop our latest version, DeepBiomarker2 by refining the relative contribution analysis for the identification of important features and integrating multiple SDOH parameters in our model. We then applied DeepBiomarker2 to PTSD outcome prediction, provided refined results specific to high-risk cohorts, proposed new interdisciplinary hypotheses and identified risk/protective medications for prevention of PTSD developing to ASUD. With the inclusion of such multimodal information, we anticipate that health professionals can improve the classification of patients based on their complexity and heterogenicity, develop targeted interventions to fulfil various clinical needs, transform health care by providing integrated services, build strong community partnerships with other health care providers for better health outcomes and reduce existing health disparities at lower costs.

## 2. Methods

### 2.1 Data source

We included data from January 2004 to October 2020 from the Neptune system at the University of Pittsburgh Medical center (UPMC) which houses EMR from the UPMC health system for research purposes (rio.pitt.edu/services). The database includes multimodal information: demographic information, diagnoses, encounters, medication prescriptions, prescription fill history, and laboratory tests. We used the ASUDs after the diagnosis of PTSD. The PTSD and ASUD patients were identified byICD9/10 codes (‘309·81’, ‘F43·10’, ‘F43·11’ and ‘F43·12’) and by ICD9/10 codes (See Appendix A) respectively^15, 16^ The medication fills include medications that a patient had filled at commercial pharmacies, collected and reported by the clearing house SureScripts. The EMR data processing steps are rigorous and hence the chance of data missing is low.

### 2.2 Data preparation

The data preparation was done in a similar fashion to that described in our previous paper DeepBiomarker^17^: For a given PTSD patient without previous diagnosis of ASUD at an index date, our primary aim was to predict whether the patient will experience ASUDs within next 3 months. In our study, a case was defined as a PTSD patient who had a record of ASUD within the next 3 months, while a control was defined as a PTSD patient with no records of ASUD in the next 3 months after the index date. The index date can be any encounter date after the PTSD diagnosis but before the first diagnosis of ASUD. If a patient had multiple encounters satisfying the inclusion criteria, we only considered the latest encounter to mimic the latest status of the patient. We excluded patients that had the diagnosis of PTSD and ASUD in the same day or experienced ASUD before PTSD diagnosis. The patient was also required to have no record of ASUD within the period of one year before the index date to negate the possibility of previous history of ASUD. We used multimodal information such as diagnosis, medications, and lab tests result 1 year preceding the index date and SDoH information at the index date as the input. We specifically included lab test results that had high frequencies and were coded as abnormal by searching results that were labeled as “ABNORMAL”, “HIGH” or “LOW”. We did not consider lab test results with low frequency (less than 100 times). We clustered diagnosis codes to diagnosis groups based of the first 3 letters of their ICD-10 codes. Medication names were converted to their respective unique DrugBank IDs. And lastly, for each encounter the associated multimodal information: diagnosis, medication and lab tests were packed into a sequence based on their respective disease categories, DrugBank IDs and lab test IDs.

### 2.3 Dataset splitting

We split our dataset with a ratio of 8:1:1, where 8 subsets were used as the training dataset, while one subset was used as the validation dataset to find the optimal parameters and the last 1 subset was used as the test set to evaluate the generalization of our model.

### 2.4 SDoH data

For each PTSD patient, we included both the individual level SDoH data and neighborhood level SDoH data. Individual level SDoH features such as race, age and gender were extracted from the demographic information in EMR and coded similar as diagnosis codes to input in the models. We also used neighborhood level SDoH features (see appendix) such as racial segregation, neighborhood socio-economic status, percentage of non-citizens, person of color index, normalized difference vegetation index, aridity index, percentage of foreign born residents, percentage of male widowers, percentage of US citizens, percentage of households with limited English proficiency, income segregation, percentage of population with same sex marriage, urban index, percentage of population who are separated from their partner, percentage of population who is a single parent, and percentage of households with transportation barriers were calculated using their respective formulas, extracted from the American community survey (ACS) (See supplementary information about SDoH parameters). Neighborhood level SDoH factors are geographically derived neighborhood level SDoH parameters that can be used for assessment of health care utilization^18^. The ACS is a rolling survey of the US population that gathers information, such as ancestry, educational level, income level, language proficiency, migration status, disability status, employment status, and housing characteristics, across 1298 variables^19^. The ACS releases estimates at the regional, state, and county level every year, and data at the census-tract, block-group and zip code levels are available every 5 years. For our study, a patient’s zip code-5 codes at the index date were used to identify their SDoH parameter. Previous studies have used all of the above indexes to represent a geographical area–based measure of the socioeconomic deprivation experienced according to their neighborhood^20^. Collectively, the data was mapped and later used as input in our model.

### 2.5 DeepBiomarker2

We adopted the Pytorch_EHR framework established by Zhi Group where Deep learning models based multiple recurrent neural networks^21^ were used to analyze and predict clinical outcomes^22^. In conjunction with their algorithm and our previous DeepBiomarker model, we further modified the framework as DeepBiomarker2 by (a) integrating individual lab tests, SDoH parameters and medications along with the diagnosis groups as the input, so that we can assess the important clinical and non-clinical factors associated with ASUD risk; and (b) refining contribution analysis^23^ module by refining the relative contribution analysis for the identification of key factors (see below for more details). Contribution analysis is a method used to calculate the relative contribution of key factors which are identified by determining the observed change in the model.

In our study, we followed the same parameters as our previous versions: embed dimension: 128; hidden size: 128; dropout rate: 0·2; number of layers: 8; input size: 30000; patience: 3. To estimate the standard deviations of the accuracy, we repeated our calculations ten times for each of the algorithms.

### 2.6 Statistical analysis

#### Assessment of importance of the clinical factors for predicting ASUD events

In order to examine the importance of the clinical factors for prediction of ASUDs, we calculated the relative contribution (RC) of each feature on the ASUD^23^. The RC of a feature was calculated as the average contribution of the feature to events divided by the average contributions of this feature to no-events. The contributions were estimated by a perturbation-based approach^24^. The equation is shown as follows where FC represents the feature contribution:

$$RC=\frac{\frac{1}{m}\sum FC_{\text{withevent}}}{\frac{1}{n}\sum FC_{\text{withoutevent}}}$$

FC value was the total value of the feature within the same patient if the feature appeared more than once in that patient, where m and n are number of patients with and without an event, respectively. The natural logarithmic form variance for RC was calculated as:

$$\text{Variance }(ln(RC))=\frac{{\left(\frac{\text{sdofFCofpatientswithevent}}{\text{meanofFCofpatientswithevent}}\right)}^{2}}{\text{numberofpatientswithevent}}+\frac{{\left(\frac{\text{sdofFCofpatientswithoutevent}}{\text{meanofFCofpatientswithoutevent}}\right)}^{2}}{\text{numberofpatientswithoutevent}}$$

sd: standard deviation

Thus, the 95% confidence interval (CI) of RC was given by:

$$95\%CI=e^{(\text{ln}(RC)\pm 1.96\sqrt{\text{Variance}(Ln(RC))}}$$

And the p-value was under the assumption of z distribution^25^. Bonferroni correction^26^ was used to reduce the type I error caused by multiple comparisons. The False discovery rate (FDR) adjusted p value is the ratio of the number of false positive results to the number of total positive test results^27^.

We improved our assessment by normalizing the FC value and scaling the RC value for all our features. The improved FC formula is the ratio of the summary of the contribution of a feature and the summary of the contribution of all the features. Next, we performed scaling, where the RC for a PTSD diagnosis was scaled to 1 and the factor generated was multiplied to get the final RC value for each of the other features. FC normalization accounts for heterogeneity in the numbers of encounters across patients, which may otherwise inflate the contribution of features observed in patients with higher healthcare utilization.

#### Assessment of model performance

The model performance was evaluated by the area under the ROC curve (AUROC), we used both deep learning and logistic regression to compare the performance of our model.

## 3. Results

### The performance of DeepBiomarker2 on the ASUD prediction

We identified 38,807 PTSD patients from UPMC EMR data. And we further identified 7,927 cases and 7,685 controls from patients with more than 1 year of EMRs before the diagnosis of PTSD (Supplementary Fig. 1). Those samples were split to 8:1:1 ratio for training, validation, and test sets. The performance metrics of the DeepBiomarker2 can be found in Table 1.

As shown in Table 1, Deep learning models: TLSTM and RETAIN algorithms implemented in DeepBiomarker2 all showed excellent performance on ASUD prediction, i.e., all yielded an AUC ≥ 0·90. The performance of deep learning (AUC above 0·93) was better than LR (0·85). The performance of models with SDoH are slightly better than those without SDoH.

### 3.1 Important indicators for the ASUD prediction

As we described previously, we followed a perturbation-based estimation approach to calculate the relative contribution of each feature on the prediction of ASUD (Supplementary table 1). Table 2, Table 3, Table 4 and Table 5 enlist the top important abnormal lab tests, medication use, diagnosis and SDoH parameters respectively. We can see that in Table 2, HGB with RC of 1·305, along with other abnormal lab tests with RC > 1 are risk factors for ASUD. In Table 3, pain medications such as acetaminophen and oxycodone both have an RC of RC > 1 which are categorized as risk factors for ASUD while medications such as oxybutynin and cetirizine have an RC < 1 and are categorized as protective factors for ASUD. In Table 4, diagnosis such as routine lab examinations are categorized as protective factor for ASUD (RC = 0·53) while emergency visit is categorized as risk factor for ASUD (RC = 1·459).

### 3.2 Overall lab test-based indicators of comorbidities and disease burdens for ASUD prediction

Through further analysis on the DeepBiomarker2 model, we identified top important lab tests as the biomarkers. Depending upon the type of biomarkers they are classified into blood, kidney, metabolic syndrome, inflammatory, urine and miscellaneous (Supplementary table 2). These biomarkers are strongly correlated to ASUD and PTSD along with its implications on adjoining diagnosis and medication use. These lab tests are indicators of underlying comorbidities and thus can be considered as measurements of disease burdens. It is not a surprise that the lab tests increase the risk of ASUDs (RC > 1, see Table 2).

## 4. Discussion

We improved our deep learning model DeepBiomarker2 to predict the risk of ASUDs in PTSD patients based on the abnormal results of regular lab tests in the last one year together with the diagnosis and medications used in the same period as well as SDoH parameters. The model yielded very good performance with AUC score above 0·93. The improvement might be from the fact that DeepBiomarker2 can also consider the sequential information of these multimodal features. To further refine our understanding of specific biomarkers pertaining to PTSD and ASUD, we have categorized our top biomarkers based on their types:

### 4.1 Biomarkers closely related to PTSD and ASUD

#### Inflammatory-based biomarkers.

We have identified two inflammatory-based biomarkers potentially useful for assessing the risks of ASUDs in PTSD patients. Current research has emphasized the importance of incorporating inflammatory biomarkers in risk prediction models to further catapult mental disorder research efforts. Several studies showed PTSD patients had elevated levels of WBC and neutrophil levels in their system on account of the activation of multiple inflammatory pathways. Abnormal WBCs and neutrophil levels express tissue function and release pro-inflammatory and pro-coagulant molecules promote thrombus formation, including platelet activation and adhesion which potentially increase the risk of cardiovascular disease in these patients^28^. Another study found low WBC and neutrophil levels in alcoholics but higher WBC levels in cannabis, inhalants, tobacco, opioid users, and no significant levels of WBCs amongst cocaine users. While low neutrophil levels were seen in cannabis users and high levels in inhalants and opioid users^29–33^.

#### Heme-based biomarkers.

The PTSD patient population might have pathologies that are related to hematopoiesis, inflammation, endothelial function and coagulability depending upon abnormal levels seen in these patients^34^. Current epidemiological studies have shown RBCs may interact with the inflammatory system and platelets. Once, exposed to oxidative stress, they acquire a senescent phenotype promoting a pro-inflammatory and pro-atherogenic state^35^. We found elevated levels of hemoglobin, red blood cells, hematocrit, RDW, MCH and MCHC in depressed patients. We also found low hemoglobin levels in alcoholics, cannabis, and heroin users^36^. While other heme biomarkers are discussed in Supplementary Table 1. To the best of our knowledge, we would be the first to propose these heme biomarkers as possible biomarkers for risk of ASUDs in PTSD patients.

#### Liver-based biomarkers.

Albumin is a protein shown to possess free radical scavenging properties that act as a selective antioxidant. There are studies examining the role of serum albumin levels in patients with psychiatric diseases^37, 38^. Current research hypothesizes that low serum albumin levels in depressed patients might be due to activation of inflammatory responses in these patients^39–41^. Another study found low serum albumin levels in drug addicts while higher serum albumin levels in alcoholics in an emergency department setting^42^. However, it is important to thoroughly examine the association between developing ASUD risk and albumin to demonstrate it as a prognostic biomarker amongst PTSD patients.

### 4.2 Effect of medication use on PTSD for ASUD prediction

#### Risk Factors:

##### Tamsulosin:

Tamsulosin is a selective α1A-adrenergic receptor antagonist which has greater cardiovascular tolerability as compared to other nonselective α-adrenergic receptor antagonists. However, recent population-based studies have shown that benign hyperplasia (BPH) patients under tamsulosin treatment have increased incidence of dementia and cognitive impairment than in the no-BPH-medication cohort^43^. The dose-response analysis done also revealed a higher risk of dementia at higher doses in the tamsulosin BPH cohort (HR, 1.12–1.49). Dementia observed in these cohorts can be attributed to two of tamsulosin properties: (1) detrimental impact on the prefrontal cortex promoting drug seeking behaviors^44^ and (2) high blood brain barrier penetration^45^ in SUD patients. Although tamsulosin has strong pharmacokinetic profiles that may suggest cognitive impairment, certain clinical studies have shown otherwise^46^. However, they may cause neuro-affective disorders such as depression and further investigation is necessary.

##### Metronidazole:

Based on the medical notes, bacterial vaginosis, esophagitis encephalopathy, and endocarditis are the popular indications for Metronidazole in these PTSD patients. PTSD has been implicated in the etiology of various gastrointestinal (GI) disorders and urinary tract infections (UTI). While multiple possible mechanisms associating PTSD and GI disorders are proposed: changes in autonomic nervous system function impact the gut-brain axis which leads to hypothalamic-pituitary-adrenal axis dysregulation and followed by abnormal cortisol levels. This along with changes in stress levels coupled with behavioral risk factors such as smoking, alcohol use and medications further exacerbate its effects^47^. Current clinical research examining the association between PTSD and GI disorders have yielded mixed evidence due to differences in methodologies of the studies. Esophagitis is one of the complications of gastrophageal reflux disease, where there is esophageal mucosal injury caused by medications. Drugs such as metronidazole damage the esophageal wall by causing a direct toxic effect on the esophageal mucosa, which produces a caustic effect by creating a highly acidic or alkaline environment^48^. The pathogenesis of UTI begins with contamination of the periurethral space by uro-pathogens such as *Escherichia coli* (E.coli) residing in the gut^49^, After this so called “intestinal bloom of uro-pathogens”, it is followed by colonization of the urethra and ascending migration to the bladder^50^. Studies have further described the gut microbiota–UTI axis where the increased abundance of *E. coli* in the gut was associated with future development of *E. coli* bacteriuria and *E. coli*-induced UTI. They found that the *E. coli* strains in the gut resembled to the *E. coli* strain in the urine, thus supporting the hypothesis that gut microbiota is a source of UTI^51, 52^. An overgrowth of gardnerella vaginalis in the genitourinary tract of women with bacterial vaginosis is associated with higher risk of sexually transmitted infections, postsurgical complications, infertility, cervical infections and cancer^53^. A case study reported a patient with Gardnerella vaginalis bacteremia with severe encephalopathy^54^. Acute encephalopathy and liver disease is another common emergency occurring most frequently due to liver failure. Cerebellar signs such as acute reversible cerebellar ataxia, dysarthria, gait instability, confusion, seizures, vertigo, dizziness, and lateralizing signs due to prolonged metronidazole use has been reported as a cause of encephalopathy^55–57^. However, the underlying mechanisms that promote the development of encephalopathy is unknown. Certain mechanisms postulated by other studies include: (1) passing the blood brain barrier and achieving therapeutic concentrations in the cerebrospinal fluid^58^, (2) binding of metronidazole and its metabolites to ribonucleic acid (RNA) in neurons which exacerbates degeneration of adjoining axons^59^, (3) generation of superoxide radicals leading to myelin oedema and vascular spasms, mild localized ischemia, mitochondrial dysfunction^60^, and (4) modulation of gamma aminobutyric acid (GABA) receptors within the vestibular and cerebellar systems^61^.

#### Cephalexin

Adverse neuropsychiatric effects from antibiotic medications are well documented. A study showed a direct relationship between acute psychosis and cephalexin exposure amongst patients with urinary tract infections. While the mechanisms for cephalexin remain unclear^62^. Potential hypothesis includes direct effect of antibiotics on neurotransmitters and their receptors, as well as anti-inflammatory effects that may modulate cytokine production and impact neurotransmitter function exerting GABA-A antagonistic effects, which potentiate excitatory activities^63, 64^..

#### Pain medications

PTSD patients prescribed with pain medications such as acetaminophen, oxycodone, hydrocodone, and gabapentin^65^ have significantly higher PTSD symptom severity scores, with opiate analgesics use associated with the highest scores^66^. The rationale was associated with use of both opiate and non-opiate analgesics leads to the hypothesis suggesting the dysregulation of the opioid system in both PTSD and ASUD. While physical injury at the time of PTSD accounts for ongoing pain symptoms seen in these patients, there is a possibility that emotional and social impact of these traumas may further exacerbate ASUD risks amongst these patients.

#### Protective factors:

##### Oxybutynin

Hyperhidrosis is a somatic disorder that involves excessive perspiration due to hyperfunctioning of sweat glands via muscarinic receptor stimulation. It is often triggered by external stimuli such as emotional, thermal, stress and physical situations^67^. Studies showed that overproduction of sweat is due to a dysfunction of the autonomic nervous system fibers caused due to clinical manifestations such as patient’s age, family history, symptom onset, co-morbidities, and medication use. Systemic disorders such as PTSD, alcoholism, traumatic brain injury, Parkinson’s disease, and diabetes can impair thermoregulatory homeostasis of the body^68^. A recent study found that patients with hyperhidrosis had experienced improvements in anxiety, depression and sweating when treated with antimuscarinic agents such as oxybutynin^69^. Another case study showed patients experiencing methadone-induced hyperhidrosis when treated with oxybutynin had improved quality of life^70^.

##### Clindamycin

Based on the medical notes, we found that endocarditis, pneumonia, and osteomyelitis are the popular indications for clindamycin in these PTSD patients. Postoperative and posttraumatic infections of bones and joints are one of the most common complications in the field of medicine. Osteomyelitis is a condition in which patients experience inflammation in the bone and bone-marrow. This could be either due to tuberculosis, syphilis, bacterial, fungal or parasitic (toxoplasma gondii) in origin^71^. There is a growing interest in epidemiology, where infections are implicated as a novel risk factor for the development of multiple mental disorders. The infection caused by neurotropic parasite Toxoplasma gondii (T. gondii) is transmitted to a host (e.g. rodent or human), via ingestion of tissue cysts in undercooked meat or oocytes in cat feces or contaminated soil, where it progresses to form focal brain lesions as seen in patients with acquired immune deficiency syndrome (AIDS)^70^. This can further lead to seizures, mental confusion, neurological impairment, ataxia, visual abnormalities, cranial nerve palsy, alcohol related dementia and psychomotor or behavioral alterations among patients with multiple etiologies^72^. A study examined the association between *T. gondii* infection, anxiety, PTSD, and depression among individuals in a population-based study. They found that seropositive individuals had more than twice the odds of reporting anxiety compared to seronegative individuals, suggesting a relationship between the immune response to *T. gondii* and other multiple anxiety and mood disorders^73^. A case study found, clindamycin treatment provided clinical improvement within 48 hours of treatment and resolved irregular brain lesions in the right basal ganglia within 3 weeks of treatment^74^. Another case study, proposed clindamycin to be used to improve cognitive function of AIDS patients with cerebral toxoplasmosis and alcohol abuse^75–77^. Thus, the use of clindamycin can be extended to patients with PTSD and ASUD.

Recent studies have suggested no major improvement in community acquired streptococcus pneumoniae meningitis. The long-term neurological sequelae coupled with its high mortality impact overall quality of life. This can be attributed to: systemic inflammatory response of the host leading to leucocyte extravasation into the subarachnoid space, brain oedema, secondary ischemia and vasculitis, stimulation of resident microglia in the central nervous system by bacterial compounds and finally interaction with the bacterial hemolysins on neurons^78–80^. A study done in a rabbit model found that clindamycin was found to pass the blood brain barrier and provided neuroprotection as opposed to other drugs in question. The proposed mechanism of action is attributed to reduced hydroxyl radical formation and lower concentrations of glutamate and glycerol in the interstitial fluid of the hippocampal formation which finally leads to decreased neuronal injury in the dentate gyrus^81^. As mentioned,, the role of dentate gyrus in bipolar disorder^82^, schizophrenia^83^ and PTSD^83^ reiterates the possibility of clindamycin’s protective effects.

##### Magnesium

Magnesium is a common dietary supplement that is taken in either as an oxide, citrate, carbonate, and sulfate forms^84^. They can be used to reduce addiction to different substances. Their ability to reduce the intensity of addiction to different substances is essentially attributed to its ability to produce a moderate stimulation of the brain reward system and its capacity to reduce the activity of glutamatergic substances, that are directly involved in compulsive use disorders^85^. Studies have shown that the drug targets the N-methyl D-aspartate (NMDA) receptors and reduces the intensity of dependence on different substances^86^. This in turn arrests reinforcement activity which is essential in compulsive drug taking behavior seen in these patients^87^.

##### Cetirizine

Allergic rhinitis is a relatively common disorder in the general population, as is asthma and also seen in patients with bipolar disorder^88^. They impact the patient’s overall social, occupational, and personal functioning. Histamine causes smooth muscle contraction, increased secretion of mucus, increased vascular permeability leading to mucosal edema, and parasympathetic nerve stimulation in these patients. In case of asthma, histamine levels are elevated due to increased peripheral basophil and mast cell degranulation, which further causes bronchospasm and hyperactivity^89^. Cetirizine is a H_1_-specific receptor antagonist that inhibits allergen-induced eosinophil chemotaxis. While, it may not have effects on serotonin, dopamine, α_1_, and muscarinic receptor. There is a possibility that H1R antagonists can cross the blood–brain barrier (BBB) and improve the quality of sleep amongst patients with major depressive disorder (MDD)^90^. Since, no study of cetirizine on the PTSD and ASUD cohort, it would be interesting to see its impact of ASUD risk among PTSD patients.

##### Montelukast

Montelukast, an anti-asthmatic drug is a leukotriene receptor antagonist (LTA) that counteracts inflammation and other detrimental pathways that are linked with other neurodegenerative diseases and allergic rhinitis may be protective or risky in nature^91^.The evidence for adverse neuropsychiatric outcomes associated with LTAs is inconclusive. Current controlled clinical trials have reported mild to infrequent adverse effects, which is indicative of montelukast being a safe and well tolerated medication. Montelukast improved learning and memory functions, followed by reduced neuroinflammation, restored blood brain barrier integrity and enhanced neurogenesis^92^. However, some case studies have reported positive dechallenge-rechallenge association, where in the adverse symptoms have resolved after treatment termination and returned after restarting the regimen^93–95^. Furthermore, since current evidence is not based on PTSD and ASUD adult population, it is essential to understand its impact on the brain circuitry amongst this subset.

###### Venlafaxine:

Certain PTSD components are attributed to stress-induced increases in the noradrenergic activity; however, the pathophysiology of PTSD pertaining to the brain’s noradrenergic pathway is underrecognized^96^. Venlafaxine is a serotonin norepinephrine reuptake inhibitor, that has demonstrated efficacy in patients with depression, generalized anxiety disorder, social anxiety disorder, and panic disorder^97^. Our findings are consistent with several other randomized clinical trials for PTSD pharmacotherapy which include: sertraline^98^, paroxetine^99^, fluoxetine^100^ and venlafaxine^100^. However, a recent clinical practice guideline study suggested that patients with depression and cannabis use, should not be treated with venlafaxine^101^. But the need for more definitive larger, multisite, randomized clinical trials is essential.

We also found that “bundled screening” for early detection and treatment of mental disorders, ASUDs, history of childhood obesity, childhood trauma and other unspecified conditions could be used as a precautionary protective factor because it can help in overall healthcare cost reduction, disseminate complications from co-occurring disorders, and overcome lack of adequate behavioral health infrastructure to provide appropriate diagnostic follow up. Other protective factors such as screening for malignant neoplasms of the cervix amongst PTSD, ASUD patients will help identify high risk patients early on. Our results are in line with a study that applied the Health Belief Model and trauma-informed frameworks to guide their analysis. They found that discomfort with pap screening was common amongst women experiencing PTSD, ASUD, homelessness and who had a history of sexual trauma such as interpersonal violence, incarceration, discrimination, and neglect^102^. Providers suggested an aggressive application of a trauma-informed approach where educating, counseling, and privacy may help address complex barriers among women experiencing PTSD, ASUD and other discomforts.

### 4.3 Effect of SDoH on PTSD for ASUD prediction

#### Negatively correlated:

##### Racial segregation

We found that patients who had values closer to −1 have higher incidences of ASUD risks as opposed to patients belonging to 1. Our results are in line with a study that showed patients belonging to heavily segregated low income areas experiencing place-based health disparities which often arise due to a results of historical segregationist policies tend to have higher levels of inflammation that were attributed to higher incidences of mental disorders^103^.

Other SDoH parameters such as low neighborhood social-economic index (nSES index), females, younger patients, black majority zip codes, low normalized difference vegetation, low aridity, zip codes with higher number of USA citizens, USA born patients, number of households with limited English speaking capacity, number of widowed partners who are males and patients belonging to zipcodes with higher income segregation all were found to have an association with PTSD and ASUD both in our study and in mental health literature^104–118^ and thus should be taken into account when reforming the health care system to respond to the challenge of health disparities.

#### Positively correlated to ASUD risk:

##### Transportation barrier

Transportation barriers due to lack of access to transportation leads to rescheduling conflicts longer wait times and missed or delayed care^119^. These in turn promote poorer management of chronic illness and mental health outcomes^120^. We found that patients living in zip codes with lack of available transportation tend to experience higher incidences of ASUD risks as opposed to patients living in zip codes with access to transportation. Our study is in line with a study which showed 5.8 million individuals in 2017 delayed medical care because they did not have access to transportation. The study emphasized that transportation barriers increased between 2003–2009 with people of color, those living below the poverty threshold, Medicaid recipients, and people with disabilities had greater odds of reporting a transportation barrier^121^. Another study found, unwillingness to be in treatment, financial/insurance, and transportation barriers to be the most common barriers to after care treatment for alcohol and substance use^122^.

Other SDoH parameters such as patients belonging to zip codes with households with same sex marriages, higher urban population and higher number of patients who are white through EMR information were found to exhibit elevated incidences of PTSD, suicide related events and ASUD^123–127^.

We also noticed that by adding neighborhood level of SDoH parameters, the performance of Deepbiomarker2 has a slightly improvement, and this might imply that some impact of those SDoH parameters have been captured by EMR data, such as medication use, health service access, etc. As shown in Table 4, routine general medical examination at a health care facility, screening for malignant neoplasms of cervix and screening for unspecified condition are protective factors with reduced ASUD risk.

### 4.4 New hypothesis on ASUD in PTSD

Most prior PTSD and ASUD studies share a common shortcoming of being rooted in the *de facto* assumption that ASUDs may emerge majorly due to biological factors. However, we would like to propose some unique hypotheses that based of our results and validated by literature. This may serve as important novel interdisciplinary indicators of mental health diseases. There exists a possible indirect link between gut microbiota dysbiosis, UTIs and Toxoplasmosis gondii^128^.Microbial dysbiosis is a major perpetrator of intestinal inflammation which promotes subsequent permeability of the gut barrier ultimately leading to distal consequences of Toxoplasmosis Gondii infection to permeate into the blood brain barrier^129^. This compromise may promote cognition and AUD risk in patients with PTSD, depression, epilepsy, suicidal ideation, GAD, and schizophrenia^130^. This health disparity can be potentially corrected by improved water purification for all. Serum albumin (SA) is closely related to oxidative stress and antioxidant capacity. It may exist in relatively high concentrations, in patients with liver disease and other neurodegeneration disorders and significantly lower in patients with cancer, critically ill patients and patients with neuropsychiatric disorders such as: schizophrenia and depression^131^. It is possible that albumin may aggravate oxidative stress by increasing the percentage of free radicals and oxidative damage products by entering the blood brain barrier. This in turn may promote inflammation while simultaneously decreases omega-3 polyunsaturated fatty acids, magnesium, thyroid hormones levels causing depression^132–135^. Another unconventional hypothesis that could be extrapolated and used for future studies is correlating allergies to PTSD and ASUD. A study showed immunoglobulin-E and WBCs to be associated with worsening of depressive scores in bipolar patients during high pollen seasons. Also, PTSD patients with nicotine dependence and chronic obstructive pulmonary disorder (COPD) had high RBCs^136^. Based on our results and literature, we show that activation of inflammatory mediators due to asthma and allergy rhinitis may be a potential biomarker for predicting ASUDs in PTSD, but further investigations are necessary.

### 4.5 Biomarkers for personalized treatment for PTSD to reduce the risk of ASUD

Using EMR and non-EMR data we examined multiple biomarkers with respect to patient medication history, diagnosis, lab tests and SDoH (both neighborhood and patient level) to propose novel therapies for PTSD patients with ASUDs. Our findings provided compelling evidence that suggests apart from targeting risk and protective factors, developing prevention and intervention strategies, one must incorporate SDoH to ameliorate sources of psychological and psychosocial risk. This in turn would help to better predict ASUD risks in PTSD patients. Although literature focuses on using conventional SDoH parameters such as income, race, unemployment to be the major predicting factors for future ASUDs, inclusion of future SDoH- ASUD factors such as peer substance use, easy drug access, low access to ASUD help centers and other psychosocial protections should be considered to obtain a comprehensive view about PTSD and SDoH. Our tool is intuitive and could build novel approaches that would provide easy interpretation of these complex biomarkers that could be applied to other future SDoH parameters. Our study is superior because of its multi-faceted data driven approach, sample size, sequential effect consideration, convenience, low cost, a suitable choice of routine testing, inclusion of SDoH and improved practical applications in any clinical setting. Unlike current biomarker studies, we considered all EMR (both clinically and non-clinically applicable) biomarkers and non EMR (non-medical and non-behavioral precursors of health) biomarkers in our study and ranked the most applicable biomarkers based on their normalized scores. Our goal is to address screening barriers, eliminate existing health disparities, provide follow-up treatment options, educate, and create support essentials to keep high risk patients linked to care.

## 5. Limitation of our study

Our study also has a few limitations: First, there could be inconsistencies of biochemical test results between patients due to enrollment bias and some lab tests might have low representation in our database. As such, the analysis might have limited power to detect the effects. Second, we used EMR data from January 2004 to October 2020, and in this period, there is a possibility of changes in treatment and number of lab tests amongst these patients. Therefore, we cannot make causal interpretations based on this. However, these are limitations caused due to using observational EMR as a data source and can be resolved by investigations using a randomized clinical trial or prospective design. Third, we considered the effect of biomarkers along with diagnosis and medication use, however in our results comorbidities had a higher impact compared to biomarkers. This can be explained since diagnosis considers the past status of the patients while biomarkers take into consideration the recent status of PTSD and ASUDs. Fourth, there are SDoH data inconsistencies due to missing data. Fifth, due to data limitations, we only mapped neighborhood level SDoH parameters based on zip codes of patient information extracted from EMR data, we did not include individual level SDoH (e.g., income, home address, education level) due to patient protection and privacy issues. Sixth, we did not include formal diagnosis by screening data but used DSM-5 diagnosis codes and due to discrepancies associated with DSM-5 diagnostic coding, there is a chance of inappropriate use of coding as seen in real practice. Our future studies will seek to find remedies to the above shortcomings by using multiple data sets, improving our algorithms, including more insightful biomarkers, and integrating other forms of metadata and other biological measures relevant to ASUD risk and performing causal pathway analyses.

## 6. Conclusion

Our improvised data driven deep learning approach aimed to identify and examine biomarkers for the assessing the risk of ASUDs among PTSD patients, which can be utilized for developing novel interdisciplinary hypothesis surrounding its etiology. Extrapolating our results and current information, we found medications like oxybutynin, magnesium oxide, clindamycin, cetirizine and montelukast all to have a potential to reduce risk of ASUDs among PTSD patients. That, being said we also found multiple SDoH parameters both conventional and unconventional ones to have significant contributions in ASUD risk prediction. The high accuracy of DeepBiomarker2 showed ASUD risk to be particularly higher among a subset of patients who may possibly experience elevated psychosocial risk coupled with pain medication treatment. This might provide a novel insight to our understanding of PTSD with ASUD in a more holistic manner. While universal prevention programs may offer current benefits, these findings from DeepBiomarker2 offer potentially valuable and refined information that can be used to design and develop personalized prevention and intervention programs that are designed to address the psychosocial needs and health disparities existing amongst these high-risk patients.

## Figures and Tables

**Figure 1:**
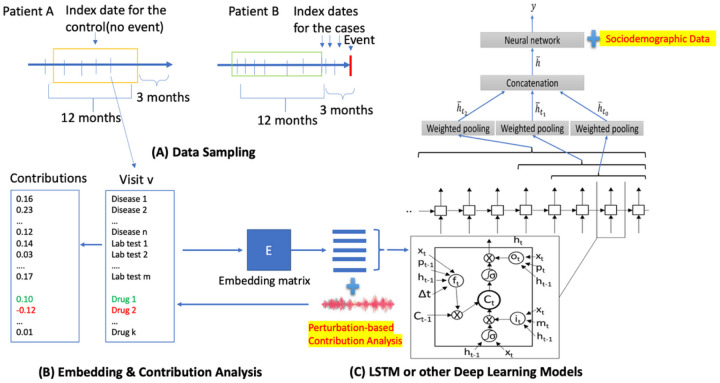
The overview of DeepBiomarker2. (A) Data sampling from electronic medical records, (B) Data embedding, and (C) Prediction by neural networks such as LSTM as the basic prediction units. Perturbation-based contribution analysis will be used to identify important features. LSTM: Long Short-Term Memory; Dx: diagnosis.

**Table 1:** The performance metrics of DeepBiomarker2 with different deep-learning and machine learning algorithms and with and without SDoH features.

RETAIN(+SDoH)	1	2	3	4	5	Average	std.s
**Validation AUC**	0.927	0.912	0.921	0.923	0.926	0.922	0.063
**Test AUC**	0.927	0.919	0.922	0.928	0.924	0.924	0.004
**Validation Precision**	0.905	0.877	0.893	0.871	0.902	0.890	0.015
**Validation Recall**	0.850	0.858	0.858	0.887	0.847	0.860	0.016
**Validation F1**	0.877	0.867	0.875	0.879	0.873	0.874	0.004
RETAIN(-SDoH)	1	2	3	4	5	Average	std.s
**Validation AUC**	0.930	0.934	0.934	0.923	0.932	0.930	0.004
**Test AUC**	0.917	0.920	0.922	0.921	0.925	0.921	0.003
**Validation Precision**	0.894	0.914	0.889	0.901	0.877	0.895	0.014
**Validation Recall**	0.852	0.853	0.867	0.844	0.873	0.858	0.012
**Validation F1**	0.872	0.882	0.878	0.871	0.875	0.876	0.004
LR(+SDoH)	1	2	3	4	5	Average	std.s
**Validation AUC**	0.860	0.868	0.862	0.869	0.8716	0.866	0.005
**Test AUC**	0.847	0.850	0.838	0.847	0.8475	0.846	0.005
**Validation Precision**	0.710	0.709	0.686	0.784	0.6924	0.716	0.039
**Validation Recall**	0.877	0.878	0.889	0.800	0.9119	0.871	0.042
**Validation F1**	0.784	0.785	0.774	0.792	0.7871	0.785	0.006
LR(-SDoH)	1	2	3	4	5	Average	std.s
**Validation AUC**	0.860	0.8561	0.857	0.862	0.856	0.858	0.003
**Test AUC**	0.845	0.8328	0.833	0.844	0.824	0.836	0.009
**Validation Precision**	0.703	0.6903	0.742	0.704	0.713	0.710	0.019
**Validation Recall**	0.891	0.8805	0.805	0.875	0.814	0.853	0.040
**Validation F1**	0.786	0.7739	0.772	0.780	0.760	0.774	0.010
TLSTM(+SDoH)	1	2	3	4	5	Average	std.s
**Validation AUC**	0.940	0.940	0.932	0.938	0.927	0.936	0.006
**Test AUC**	0.925	0.924	0.925	0.932	0.922	0.926	0.004
**Validation Precision**	0.836	0.846	0.829	0.880	0.828	0.844	0.022
**Validation Recall**	0.899	0.892	0.907	0.862	0.882	0.888	0.018
**Validation F1**	0.867	0.868	0.866	0.871	0.854	0.865	0.007
TLSTM(-SDoH)	1	2	3	4	5	Average	std.s
**Validation AUC**	0.932	0.926	0.933	0.918	0.936	0.929	0.007
**Test AUC**	0.932	0.925	0.931	0.930	0.927	0.929	0.003
**Validation Precision**	0.873	0.857	0.843	0.879	0.882	0.867	0.016
**Validation Recall**	0.897	0.884	0.904	0.865	0.867	0.883	0.018
**Validation F1**	0.885	0.871	0.873	0.872	0.874	0.875	0.006

* AUC: area under curve; std: standard deviation, TLSTM: Tan Long Short-Term Memory; RETAIN: Reverse Time AttentIoN model; LR: Logistic regression

**Table 2:** Top important abnormal lab test results identified by perturbation-based contribution analysis for ASUD prediction.

Feature Name	Relative Contribution	CI95up	CI95down	FDR_Q	p_bonferroni
HGB	1·305	1·374	1·239	9·25E-20	6·47E-19
HEMATOCRIT(HCT)	1·292	1·362	1·225	1·08E-17	1·08E-16
PROTEIN-URINE	1·461	1·614	1·322	3·83E-11	9·58E-10
GLUCOSE	1·193	1·253	1·137	2·99E-10	9·28E-09
RDW	1·240	1·317	1·167	6·88E-10	2·41E-08
RBC	1·202	1·272	1·137	1·81E-08	7·98E-07
WBC	1·217	1·295	1·144	6·14E-08	3·13E-06
ABS NEUTROPHILS	1·239	1·328	1·156	1·13E-07	6·57E-06
ALBUMIN	1·255	1·353	1·164	2·62E-07	1·65E-05
MCH	1·191	1·269	1·117	3·41E-06	0·00001
LEUKOCYTE ESTERASE	1·233	1·339	1·136	1·66E-05	0·002
BACTERIA IN URINE	1·218	1·320	1·123	4·91E-05	0·006
MCHC	1·180	1·267	1·099	0·0001	0·017

* Hemoglobin (HGB), hematocrit (HCT), red cell distribution width (RDW), red blood cells (RBC), white blood cells (WBC), absolute (ABS) neutrophils, mean corpuscular hemoglobin (MCH) and mean corpuscular hemoglobin concentration (MCHC). FDR_Q: false discovery rate adjusted Q value; CI: confidence Interval, p_bonferroni: P values with Bonferroni correction.

**Table 3:** Top important medication uses results identified by perturbation-based contribution analysis for ASUD prediction.

Feature Name	Relative Contribution	CI95up	CI95down	FDR_Q	p_bonferroni
Acetaminophen	1·524	1·656	1·403	2·03E-19	1·62E-18
Oxycodone	1·647	1·833	1·480	1·83E-16	2·74E-15
Oxybutynin	0·450	0·596	0·341	1·22E-06	9·04E-05
Cephalexin	1·692	2·049	1·397	3·27E-06	0·0003
Metronidazole	1·629	1·946	1·363	3·41E-06	0·0003
Gabapentin	1·290	1·423	1·169	1·41E-05	0·0014
Hydrocodone	1·331	1·489	1·190	1·68E-05	0·0018
Clindamycin	0·650	0·775	0·545	4·37E-05	0·0051
Cetirizine	0·666	0·790	0·562	6·97E-05	0·0089
Montelukast	0·689	0·806	0·589	7·17E-05	0·0094
Magnesium Oxide	0·640	0·779	0·526	0·0002	0·0249
Tamsulosin	1·750	2·255	1·358	0·0003	0·0430
Venlafaxine	0·754	0·873	0·657	0·0013	0·3176

* Relative contribution value > 1: Risk and Relative contribution value; < 1: Protective; FDR_Q: false discovery rate adjusted Q value; CI: confidence Interval

**Table 4:** Top important diagnosis results identified by perturbation-based contribution analysis for ASUD prediction.

Feature Name	Relative Contribution	CI95up	CI95down	FDR_Q	p_bonferroni
Routine general medical examination at a health care facility	0·531	0·571	0·493	2·64E-55	2·64E-55
Emergency Visit	1·459	1·530	1·391	9·95E-46	1·99E-45
Asthma, unspecified type, unspecified	1·420	1·533	1·315	7·58E-16	1·21E-14
Lumbago	1·340	1·446	1·242	2·39E-11	5·26E-10
Other chronic pain	1·283	1·391	1·183	1·35E-07	7·99E-06
Diabetes mellitus without mention of complication, type II or unspecified type, not stated as uncontrolled	1·698	2·027	1·422	3·32E-07	2·16E-05
Screening for malignant neoplasms of cervix	0·796	0·860	0·737	4·14E-07	2·85E-05
Major depressive affective disorder, recurrent episode, moderate	1·342	1·491	1·208	2·15E-06	0·0002
Long-term (current) use of anticoagulants	1·233	1·331	1·143	3·16E-06	0·0003
Other general symptoms	0·670	0·776	0·579	3·83E-06	0·0004
History of Body Mass Index, pediatric, greater than or equal to 95th percentile for age	0·776	0·857	0·703	1·85E-05	0·0020
Screening for unspecified condition	0·771	0·860	0·691	6·97E-05	0·0090
Fibromyalgia	1·183	1·275	1·097	0·0002	0·0326

* Relative contribution value > 1: Risk and Relative contribution value; < 1: Protective; FDR_Q: false discovery rate adjusted Q value; CI: confidence interval

**Table 5:** Top important SDoH results identified by perturbation-based contribution analysis for ASUD prediction.

SDoH	Mean	Standard Deviation	CIdown	CIup	FDR_Q	p_bonferroni	Impact on ASUD risk	Type of SDoH
Age	−0.117	0.038	−0.140	−0.093	−9.606	2.15E-06	Younger patients have higher risk of ASUD	Individual
Neighborhood socioeconomic status	−0.119	0.047	−0.149	−0.090	−8.000	1.11E-05	Neighborhoods with low socio-economic status has higher risk of ASUD	Neighborhood
Racial segregation	−0.106	0.042	−0.132	−0.080	−7.969	1.14E-05	High racially segregated zip codes have higher risk of ASUD	Neighborhood
Race (White)	0.155	0.069	0.113	0.198	7.160	2.88E-05	White patients have higher risk of ASUD	Individual
Aridity	−0.041	0.019	−0.053	−0.029	−6.796	4.48E-05	Low Humidity/lower vegetation/greenery have higher risk of ASUD	Neighborhood
Household with transportation barriers	0.086	0.044	0.059	0.113	6.161	0.0001	Households with no vehicles have higher risk of ASUD	Neighborhood
Household with same sex marriages	0.065	0.033	0.044	0.085	6.116	0.0001	Households with same sex marriages have a higher chance of ASUD risk	Neighborhood
Percentage of Non-Citizens	−0.097	0.055	−0.131	−0.063	−5.593	0.0002	US Citizens have a higher chance of ASUD risk	Neighborhood
Percentage of Foreign born	−0.077	0.047	−0.106	−0.048	−5.128	0.0004	US born patients have higher risk of ASUD	Neighborhood
Urban index	0.055	0.038	0.032	0.079	4.612	0.0009	Urban population have higher risk of ASUD	Neighborhood
Limited English-speaking household	−0.089	0.066	−0.130	−0.048	−4.267	0.0015	Households with limited english speaking capacity have higher risk of ASUD	Neighborhood
Gender	−0.108	0.084	−0.159	−0.056	−4.074	0.0021	Females have higher risk of ASUD	Individual
People of color index	−0.065	0.051	−0.097	−0.033	−3.990	0.0024	Black majority have higher risk of ASUD	Neighborhood
Widowed partner who is a Male	−0.090	0.082	−0.141	−0.039	−3.481	0.0055	Widowed partner who is a male have lower risk of ASUD as opposed to widowed partner who is a female	Neighborhood
Normalized difference vegetative index	−0.076	0.074	−0.121	−0.030	−3.242	0.0081	Low vegetation/greenery have higher risk of ASUD	Neighborhood
Income segregation	−0.049	0.049	−0.079	−0.018	−3.124	0.0099	Households with higher income segregation have higher risk of ASUD	Neighborhood

* FDR_Q: false discovery rate adjusted Q value; CI: confidence interval

## Data Availability

The data used in this study were from UPMC under a data use agreement. The authors are not permitted to distribute the data to any third party, but researchers may contact UPMC for data access.
